# Three cases report of gastrointestinal leaks after gastric suturing treated with self-made water-drip negative-pressure dual-lumen tube combined with enteral nutrition

**DOI:** 10.1097/MD.0000000000047848

**Published:** 2026-02-20

**Authors:** Cheng-Rong Ling, Hai-wen Ye, Cheng Tian, Jun-jiang Pan, Fei Liu

**Affiliations:** a Department of General Surgery, Second People’s Hospital of Yibin City, Yibin, Sichuan, China.

**Keywords:** enteral nutrition, gastric perforation, gastrointestinal leaks, negative-pressure double-lumen tube

## Abstract

**Rationale:**

Postoperative gastrointestinal leaks complicated by severe intra-abdominal infection remain a significant clinical challenge. This report describes a conservative management approach using a self-made, water-drip, negative-pressure, double-lumen tube combined with enteral nutrition in three cases, highlighting its potential as a technical option when immediate surgical closure is not feasible.

**Patient concerns:**

Three patients developed gastrointestinal leaks with severe intra-abdominal infection following gastric suturing. They presented with signs of peritonitis, sepsis, and persistent drainage from surgical sites.

**Diagnoses:**

Gastrointestinal leaks were confirmed postoperatively by imaging studies (e.g., contrast-enhanced computed tomography or fistulography) and clinical manifestations of severe intra-abdominal infection.

**Interventions:**

A self-made, water-drip, negative-pressure, double-lumen tube was placed percutaneously for active irrigation and drainage of the infected cavity, combined with enteral nutritional support to maintain metabolic needs and promote healing.

**Outcomes:**

Two patients achieved full recovery and were discharged. The third patient showed significant clinical improvement and was transferred to a local hospital for continued care, where they eventually recovered and were discharged.

**Lessons:**

For complex postoperative gastric leaks unsuitable for immediate definitive closure due to poor tissue quality and severe infection, percutaneous active irrigation and drainage using a self-made, water-drip, negative-pressure, double-lumen tube can be a valuable technical option within a multidisciplinary comprehensive management strategy. This approach may help control infection and facilitate healing while avoiding high-risk reoperation.

## 1. Introduction

Gastrointestinal ulcer perforation is a common surgical emergency worldwide, associated with mortality rates as high as 30% to 40%.^[1,2]^ Surgical intervention is the primary treatment for confirmed gastrointestinal perforation. Multiple studies emphasize that early intervention significantly improves prognosis.^[3,4]^ Advanced age, comorbidities, and use of nonsteroidal anti-inflammatory drugs or corticosteroids are known to adversely affect outcomes.^[5]^ Additionally, when gastrointestinal leaks occur after primary suture repair, re-suturing may not be technically feasible; in such instances, resection of the perforated site is considered a safer alternative.^[1]^ However, for patients who develop postoperative gastrointestinal leaks complicated by severe intra-abdominal infection, surgical re-intervention carries an extremely high-risk of morbidity and mortality, posing a major therapeutic dilemma.

**Figure 1.**
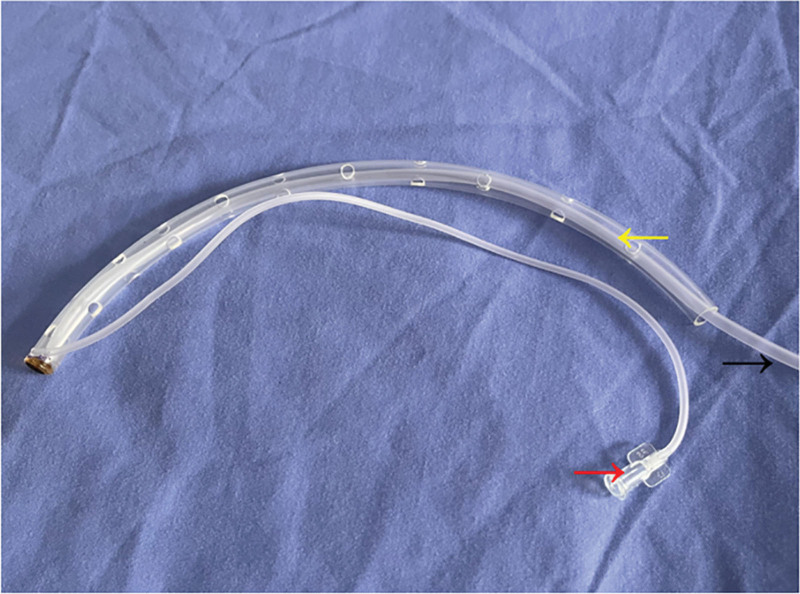
Self-made water drop negative pressure dual-chamber tube (the yellow arrow indicates the latex tube; the black arrow indicates the suction tube; the red arrow indicates the inlet tube).

We present 3 cases of gastrointestinal leak with severe intra-abdominal infection following gastric suturing, managed at our institution between August 2024 and March 2025. Conservative management using a self-made water-drip negative-pressure double-lumen tube (Fig. 1), combined with enteral nutritional support, achieved favorable clinical outcomes, offering a viable alternative for the nonoperative management of complex gastrointestinal leaks. This study was approved by the Ethics Review Committee of the Second People’s Hospital of Yibin, approval number: 2025-245-01. All patients provided informed consent for publication.

### 1.1. Data and methods

#### 1.1.1. Data

##### Case 1

**Figure 2.**
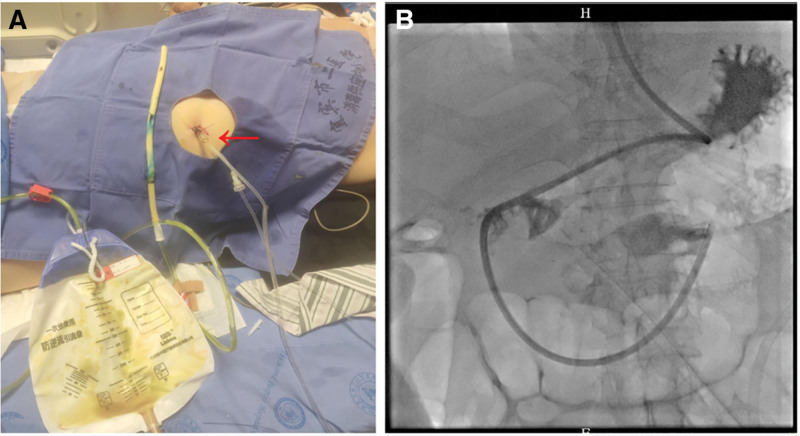
(A) Oral methylene blue flows out through the abdominal drainage tube, the arrow indicates the replaced negative pressure dual-chamber tube; (B) enteral nutrition tube.

A 59-year-old male patient with a medical history of hypertension, diabetes mellitus, and coronary heart disease and a 10-year history of gout treated with long-term oral analgesics (febuxostat and “headache powder”) was admitted due to persistent right upper quadrant abdominal pain lasting 5 days. Emergency computerized tomography (CT) suggested possible perforation of the gastric antrum or duodenum. Emergency laparoscopic exploration confirmed a posterior wall perforation of the gastric antrum, which was repaired laparoscopically. A drainage tube was placed near the repair site. Postoperatively, the patient received total parenteral nutrition and piperacillin-tazobactam. Subsequently, the patient developed recurrent high fever. Laboratory tests revealed elevated inflammatory markers: procalcitonin 71.94 ng/mL; white blood cell count: 13.20 × 10^9^/L, neutrophil percentage: 96.50%. After pharmaceutical consultation, antibiotic therapy was escalated to meropenem. Approximately 1 week postoperatively, gastric contents were drained via the drainage tube, accompanied by recurrent high fever. Oral methylene blue administration resulted in blue discoloration of the drain output (Fig. 2A), confirming postoperative gastrointestinal leak. The antibiotic therapy was adjusted to meropenem plus vancomycin. The original drainage tube was replaced with a self-made water-drip negative-pressure double-lumen tube to enable continuous suction, and somatostatin was initiated to suppress gastrointestinal secretions. Following interventional radiology consultation, a jejunal feeding tube was placed (Fig. 2B). Culture of the patient’s abdominal drainage fluid grew *Candida albicans*. The treatment regimen was then optimized to include triple antibiotic therapy (meropenem + vancomycin + fluconazole) + combined enteral nutrition (short-chain peptides via jejunal tube) and parenteral nutrition (lipid emulsion + amino acids) + esomeprazole for acid suppression + Kangfuxin + sucralfate suspension gel to promote ulcer healing + live combined *Bacillus subtilis* and *Enterococcus faecium* enteric-coated capsules restore intestinal flora balance.

##### Case 2

**Figure 3.**
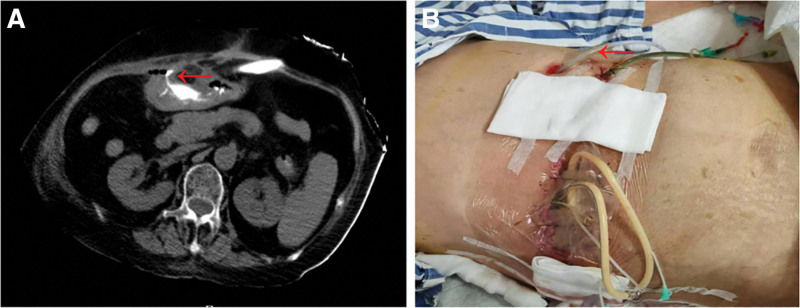
(A) The arrow indicates the leakage of the contrast agent; (B) the skin around the patient’s abdominal incision is red and swollen, showing a chemical inflammatory reaction (the arrow indicates the replaced negative pressure dual-chamber tube).

An 80-year-old female patient with a family-reported history of long-term oral analgesic use (“headache powder”) was admitted 14 days after undergoing exploratory laparotomy with antrum repair at another hospital following a gastric perforation diagnosis. On postoperative day 2, gastric contents were observed in the drainage fluid. The condition progressed to incision infection with digestive juice leaks, necessitating 3 repeated abdominal debridement and drainage procedures. As the intra-abdominal infection worsened, accompanied by secondary infections of the abdominal wall and incision, the patient was transferred to our institution. Physical examination upon admission revealed: multiple intra-abdominal drainage tubes with purulent discharge containing intestinal contents, along with peri-wound skin erythema and swelling consistent with chemical dermatitis. Emergency CT indicated fluid and gas accumulation in the upper abdomen, with encapsulated subcapsular fluid in the prehepatic space. Contrast agent extravasation was noted, suggesting postoperative gastrointestinal leak (Fig. 3A). Immediate emergency debridement was performed with placement of vacuum-assisted closure dressing, and the existing gastroduodenal drainage tube was replaced with a self-made water-drip negative-pressure double-lumen tube to enable continuous suction (Fig. 3B). Somatostatin was administered to reduce digestive secretion. The patient developed recurrent high fever (peak temperature: 41°C). Blood cultures confirmed *C albicans* infection. Following multidisciplinary consultation involving infectious disease specialists and clinical pharmacists, antimicrobial therapy was optimized to piperacillin-tazobactam plus caspofungin. The treatment regimen was further adjusted to include: combined nutritional support (enteral nutrition via jejunal feeding tube with short-chain peptides + parenteral nutrition with lipid emulsion and amino acids), esomeprazole for acid suppression, Kangfuxin and sucralfate suspension gel to promote ulcer healing, plus live combined *B subtilis* and *E faecium* enteric-coated capsules to restore intestinal microbial balance.

##### Case 3

**Figure 4.**
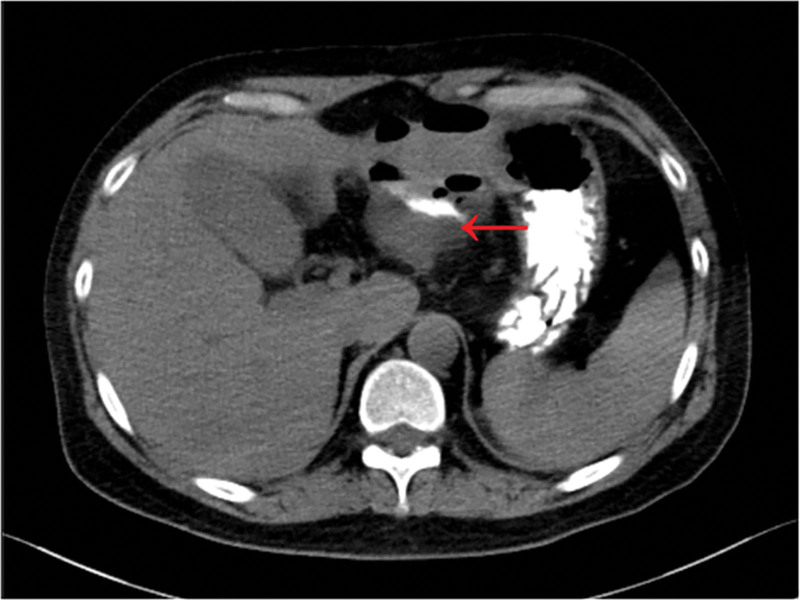
Arrows indicate the presence of fluid and gas accumulation, as well as the leakage of contrast agent.

A 38-year-old male patient with a medical history of hypertension and over 7 years of gout. The patient had been treated long-term with prednisone, colchicine, and “headache powder,” with irregular use of febuxostat for uric acid control. This admission occurred 10+ days post gastric perforation surgery. Ten days prior to admission, he underwent laparoscopic repair of a gastric antrum perforation at an outside hospital. Postoperatively, the patient was transferred to our hospital due to progressive deterioration of infection. Admission laboratory tests showed leukocytosis: white blood cell count: 22.25 × 10^9^/L, absolute neutrophil count: 19.85 × 10^9^/L, procalcitonin: 3.48 ng/mL; abdominal CT revealed thickening of the gastric antrum wall with a small ulcer on the lesser curvature, surrounded by fluid and gas collections forming a contrast-enhanced encapsulated area (Fig. 4). Adjacent peritoneal thickening, increased density, and blurred fat planes were also observed. Findings were consistent with postoperative gastrointestinal leaks. A jejunal feeding tube was placed. and the drainage system was upgraded to continuous irrigation and drainage using a self-made water-drip negative-pressure double-lumen tube. Somatostatin was administered to suppress digestive secretions. Cultures from urine and abdominal drainage fluid identified *E faecium*, while sputum culture grew *C albicans*. Following hospital-wide consultation, the treatment regimen was adjusted to: triple antibiotic therapy (piperacillin-tazobactam + linezolid + fluconazole); combined nutritional support including enteral nutrition via jejunal feeding tube (short-chain peptides, etc) and parenteral nutrition (lipid emulsion + amino acids); esomeprazole for acid suppression; Kangfuxin and sucralfate suspension gel to promote ulcer healing; plus live combined *B subtilis* and *E faecium* enteric-coated capsules to restore intestinal microbial balance.

To clarify the key therapeutic processes and their temporal relationships for each patient, a timeline summarizing interventions and outcomes is provided in Table 1, in accordance with the CARE guidelines.

**Table 1:** **The flowchart for describing 3 cases**.

Clinical stage/time course	Case 1 (59 yr, M)	Case 2 (80 yr, F)	Case 3 (38 yr, M)
Presentation and initial surgery	Clinical event: Emergency laparoscopic repair for perforated gastric ulcer. (the first day of admission) Reasoning: Perforation confirmed; primary repair deemed feasible. Drain placed for surveillance	Clinical event: Referral 14 d post open repair with persistent leak and infection Reasoning (at transfer): Established, complex leak with systemic infection. Goal: source control without high-risk immediate surgery	Clinical event: Referral 10+ d post laparoscopic repair with worsening infection Reasoning (at transfer): Failed initial management. Need to diagnose leak extent and control sepsis
Postoperative complication	Clinical event: Recurrent fever, then gastric contents were drained via the drainage tube Differential/problem: Sepsis source. Pneumonia,or intra-abdominal? Decision and rationale: Methylene blue test to confirm GI leak	Clinical event: Incisional infection, chemical dermatitis, ongoing purulent drainage Differential/problem: Uncontrolled source of intra-abdominal sepsis and locoregional tissue damage Decision and rationale: Emergency CT and debridement: To assess anatomy and clean infected wound	Clinical event: Leukocytosis, peritoneal signs on CT Differential/problem: Confirming leak or abscess Decision and rationale: CT with oral contrast: To confirm and localize leak
Targeted intervention	Decision and rationale: 1. Severe infection and poor tissue vitality make reoperation and endoscopic treatment unsuitable 2. Change the drainage 3. Upgrade antibiotics: To cover MDR organisms given high PCT and clinical deterioration	Decision and rationale: 1. Severe infection and poor tissue vitality make reoperation and endoscopic treatment unsuitable 2. Change the drainage 3. Upgrade antibiotics: To cover MDR organisms given high PCT and clinical deterioration	Decision and rationale: 1. Severe infection and poor tissue vitality make reoperation and endoscopic treatment unsuitable 2. Change the drainage 3. Upgrade antibiotics: To cover MDR organisms given high PCT and clinical deterioration
Comprehensive management phase	Interventions: Negative pressure irrigation and drainage; Triple therapy (meropenem/vancomycin/fluconazole), EN/PN, PPI, mucosal protectants, probiotics Rationale: Combined strategy to control infection, reduce secretion, promote healing, and maintain gut barrier function	Interventions: Negative pressure irrigation and drainage; Dual therapy (piperacillin-tazobactam/caspofungin), EN/PN, PPI, mucosal protectants, probiotics Rationale: Combined strategy to control infection, reduce secretion, promote healing, and maintain gut barrier function	Interventions: Negative pressure irrigation and drainage; Triple therapy (piperacillin-tazobactam/linezolid/fluconazole), EN/PN, PPI, mucosal protectants, probiotics Rationale: Combined strategy to control infection, reduce secretion, promote healing, and maintain gut barrier function
Outcome	Leak sealed, recovered	Infection controlled, gradual recovery	Leak sealed, recovered

CT = computerized tomography, EN = enteral nutrition, GI = gastrointestinal, MDR = multidrug-resistant organism infection, PCT = procalcitonin, PN = parenteral nutrition, PPI = proton pump inhibitor.

## 2. Materials and methods

### 2.1. Operational protocol and monitoring for the drainage system

The application of this self-made water-drip negative-pressure double-lumen drainage system followed these key operational protocols:

1. Dynamic adjustment of parameters: The irrigation volume and negative pressure were dynamically adjusted according to the stage of infection control. During the acute infection phase (with large fistula and severe inflammation), continuous rapid irrigation was employed, with a normal saline volume of 6000 to 9000 mL/d and negative pressure maintained at 0.04 to 0.06 MPa. In the infection improvement phase, this was switched to continuous low-negative-pressure drainage, reducing the irrigation volume to approximately 3000 mL/d and the pressure to below 0.02 MPa.
2. Scheduled drainage tube replacement: To prevent occlusion of the side holes by omental adhesions, necrotic debris, or fibrin deposits, the drainage tube was proactively replaced approximately once a week. The specific timing was individualized based on the resolution of infection and drainage patency.
3. Precise tube positioning: The distal end of the drainage tube should be positioned approximately 1 to 2 cm from the fistula orifice. This ensures effective drainage while avoiding direct contact with the damaged tissue (which could impair healing) or being placed too far away (which could reduce drainage efficacy).
4. Imaging-guided gradual tube removal: A routine oral contrast CT scan was performed prior to removal to confirm the absence of contrast extravasation and substantial control of infection. The removal process should be gradual and slow. Based on clinical and imaging assessment, the tube was withdrawn incrementally at a rate of approximately 2 to 3 cm/wk to facilitate stepwise healing of the sinus tract.
5. Close monitoring of drainage fluid: Especially in the early treatment phase, the volume and color of the drainage fluid were closely monitored. If the fluid appeared overtly bloody, the negative pressure was promptly reduced, and irrigation was temporarily suspended if necessary, with vigilance for potential hemorrhagic complications.
6. Comprehensive nursing care and complication prevention: As the treatment required prolonged bed rest, we implemented systematic nursing measures, including prophylaxis against venous thromboembolism, to address risks associated with immobility.

## 3. Results

**Figure 5.**
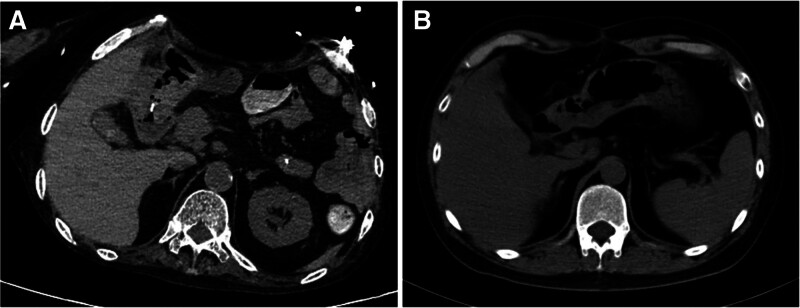
The reexamination CT scan shows clear fat tissue around the stomach, with no obvious signs of fluid accumulation or air accumulation. CT = computerized tomography.

Case 1 remained hospitalized for 89 days and was discharged with clinical improvement. Follow-up abdominal CT at 3 weeks post-discharge showed mild blurring of perigastric adipose tissue without significant fluid or gas accumulation in the abdominal cavity (Fig. 5A). Case 2 was hospitalized for 49 days; wounds healed well and oral intake was gradually resumed. Per family request, the patient was transferred to a local hospital for continued care. Telephone follow-up at 3 months post-discharge confirmed complete wound healing and full resumption of a normal diet. Case 3 was hospitalized for 47 days. Follow-up CT at 3 months post-discharge demonstrated clear perigastric fat planes with no evidence of residual fluid or gas collection (Fig. 5B).

## 4. Discussion

Gastrointestinal leak after gastric suturing refers to the persistent escape of gastric contents through inadequately healed suture lines into the peritoneal cavity, leading to chemical peritonitis and subsequent bacterial infection. Without timely intervention, this condition may progress to sepsis and multiple organ dysfunction syndrome, posing a life-threatening risk. Gastrointestinal leaks have consistently posed challenging clinical complications. Historically, surgical interventions have been the primary approach^[3,4,6]^; however, reoperation for leak management is associated with substantial mortality risks.^[7]^ Consequently, minimally invasive endoscopic techniques – such as stent placement, endoscopic drainage, suturing, and clipping – have gained increasing recognition and demonstrating promising outcomes in selected cases.^[8-10]^

Nevertheless, standardized treatment protocols for gastrointestinal leaks have not yet been established. Successful management requires individualized, multidisciplinary strategies tailored to patient-specific factors, including comorbidities, infection severity, and anatomical considerations. Such decisions must also account for institutional resources and technical expertise.^[7]^ Endoscopic therapy and surgical reinterventions depend on adequate tissue viability for closure^[10,11]^ and require advanced equipment and skilled operators. As a result, effective treatment of complex gastrointestinal leaks is largely confined to tertiary care centers, limiting accessibility – particularly in resource-limited or economically underdeveloped regions.

This report describes our preliminary experience in 3 patients with complex gastric leaks, where immediate definitive surgical or endoscopic closure was not feasible. We employed a self-made water-drip negative-pressure double-lumen tube for continuous irrigation and drainage, combined with a stepwise nutritional support strategy. Our aim is to share the clinical decision-making process and technical details within this specific context, without asserting the general superiority of this technique.

### 4.1. Reconsideration of case selection and clinical decision-making

The cases in this series exhibited significant heterogeneity in terms of age, primary surgery, and number of prior interventions. However, they shared 2 critical characteristics that formed the rationale for selecting this drainage approach. First, all patients presented with severe intra-abdominal infection and impaired tissue healing due to long-term use of nonsteroidal anti-inflammatory drugs or corticosteroids, rendering direct surgical repair or endoscopic clipping/suturing exceedingly high-risk and of low feasibility. Second, following initial surgery or percutaneous drainage, a sinus tract leading to the leakage site had developed in each case, providing a conduit for placing the customized drainage device. This scenario aligns with the concept of a “salvage” or “control” drainage strategy.^[12]^ For instance, patient 2, who underwent multiple operations including vacuum-assisted closure therapy within a short period, had an abdominal environment unsuitable for re-anastomosis, shifting the therapeutic focus to infection source control and nutritional support. The principle of our method is similar to modified negative-pressure therapy techniques reported in the literature for managing upper gastrointestinal perforations or fistulas, which aim to control contamination and promote granulation tissue formation through active drainage.^[12]^ Compared to vacuum-assisted closure systems, our apparatus is assembled from generic consumables at a lower cost, but it is applied via a percutaneous sinus tract rather than through endoscopic intraluminal placement.

### 4.2. Active drainage as a core component of comprehensive management

It must be emphasized that the clinical improvement observed in this case series cannot be attributed solely to the self-made drainage tube. Successful management was the result of comprehensive therapy, including broad-spectrum antimicrobial agents, meticulous fluid resuscitation, somatostatin analogs (to reduce digestive secretions), and, most crucially, nutritional support.^[13]^ The function of the drainage tube was to provide continuous and controllable active drainage, a cornerstone in the management of digestive tract fistulas. Traditional passive drains are prone to occlusion by fibrin or necrotic debris. Our device, by continuously infusing saline through an independent irrigation lumen and simultaneously aspirating it via a negative-pressure suction lumen, may theoretically remove leaked digestive fluids and infected necrotic material more effectively. This mechanism can mitigate chemical irritation to surrounding tissues and help establish conditions for infection control. This “irrigation-suction” mechanism shares a conceptual basis with negative-pressure wound therapy combined with irrigation for managing complex wounds.

### 4.3. Stepwise strategy for nutritional support

All patients followed a stepwise strategy transitioning from parenteral to enteral nutrition. Enteral nutrition is recognized as the safest and most physiologically appropriate form of nutritional support.^[14]^ Specialized enteral formulas containing a combination of multiple nutrients including glutamine, fish oil, arginine, and nucleotides – have been shown to promote intestinal mucosal repair and enhance immune function, improving clinical outcomes and reducing hospitalization duration in critically ill patients.^[15,16]^

Once the infection was preliminarily controlled (e.g., decreasing body temperature, trending normalization of white blood cells), we initiated enteral nutrition via a jejunal feeding tube. Early enteral nutrition helps maintain intestinal mucosal barrier function, modulates immunity, and avoids complications associated with long-term parenteral nutrition.^[13]^ The successful management in this series reinforces the importance of integrating aggressive nutritional support with infection source control under the guidance of a multidisciplinary team, including clinical nutritionists.

#### 4.3.1. Methodological considerations and limitations

This study has several important limitations that warrant cautious interpretation: This is a descriptive report of only 3 patients. The lack of a control group precludes any conclusions regarding comparative efficacy. As mentioned, the outcomes resulted from the combined effects of multiple interventions. The independent contribution of the self-made drainage tube cannot be quantified. The device is self-assembled, and its effectiveness may be influenced by operator experience. The technical parameters described (e.g., pressure adjustment, tube replacement timing, distance from the fistula) are based on the limited experience of our center and require further validation. Furthermore, although we defined fistula closure based on the absence of contrast extravasation on imaging and the resolution of clinical signs of infection, 1 case relied on telephone follow-up. Detailed long-term imaging and functional status data (e.g., quality of life) are lacking. The follow-up period was relatively short, insufficient for assessing long-term complications.

## 5. Conclusions and future directions

For complex postoperative gastric leaks unsuitable for immediate definitive closure due to poor tissue quality and severe infection, percutaneous active irrigation and drainage using a self-made water-drip negative-pressure double-lumen tube can be considered a technical option within a multidisciplinary comprehensive management strategy. This report preliminarily describes its feasibility and operational considerations in specific clinical scenarios. However, this remains an exploratory summary of experience. The efficacy, safety, and optimal parameters of this technique require validation through prospective studies with larger cohorts and comparisons with existing standard drainage methods. Future research should focus on standardizing outcome measures (e.g., time to definitive closure, re-intervention rate) and evaluating cost-effectiveness, particularly its potential value in resource-diverse healthcare settings.

## Acknowledgments

The authors would like to thank all the participants in the study, and we are grateful to all the medical staff who participated in the study.

## Author contributions

**Data curation:** Cheng-Rong Ling, Hai-wen Ye, Cheng Tian, Jun-jiang Pan, Fei Liu.

**Writing – original draft:** Cheng-Rong Ling.

**Writing – review & editing:** Cheng-Rong Ling, Fei Liu.

## References

[1] Søreide K Thorsen K Harrison EM. Perforated peptic ulcer. Lancet. 2015;386:1288–98. 26460663 10.1016/S0140-6736(15)00276-7 PMC4618390

[2] Tarasconi A Coccolini F Biffl WL. Perforated and bleeding peptic ulcer: WSES guidelines. World J Emerg Surg. 2020;15:3. 31921329 10.1186/s13017-019-0283-9 PMC6947898

[3] Lunevicius R Morkevicius M. Risk factors influencing the early outcome results after laparoscopic repair of perforated duodenal ulcer and their predictive value. Langenbecks Arch Surg. 2005;390:413–20. 16041553 10.1007/s00423-005-0569-0

[4] Surapaneni S Rajkumar S Reddy A VB. The perforation-operation time interval; an important mortality indicator in peptic ulcer perforation. J Clin Diagn Res. 2013;7:880–2. 23814733 10.7860/JCDR/2013/4925.2965 PMC3681060

[5] Møller MH Adamsen S Thomsen RW Møller AM. Preoperative prognostic factors for mortality in peptic ulcer perforation: a systematic review. Scand J Gastroenterol. 2010;45:785–805. 20384526 10.3109/00365521003783320

[6] Cereatti F Grassia R Drago A Conti CB Donatelli G. Endoscopic management of gastrointestinal leaks and fistulae: what option do we have? World J Gastroenterol. 2020;26:4198–217. 32848329 10.3748/wjg.v26.i29.4198 PMC7422542

[7] Hoffman A Atreya R Rath T Dorlöchter C Neurath MF. Endoscopic management of perforations, gastrointestinal leaks, and fistulae. Visceral medicine. 2025;41:147–58. 10.1159/000545072 PMC12052361 40330636

[8] Li Y Li Z Li Y Li Z Ren K. Successful closure of thoracogastric airway fistula with occluder devices and coils under fluoroscopic guidance. Endoscopy. 2023;55:E1075–6. 37758213 10.1055/a-2155-3369 PMC10533354

[9] Li Z Li Y Li Z. Three-dimensional printing-assisted interventional therapy for thoracogastric right main bronchial fistula. Endoscopy. 2024;56:E262–3. 38485160 10.1055/a-2274-5928 PMC10940068

[10] Matteo MV Birligea MM Bove V. Management of fistulas in the upper gastrointestinal tract. Best Pract Res Clin Gastroenterol. 2024;70:101929. 39053982 10.1016/j.bpg.2024.101929

[11] Willingham FF Buscaglia JM. Endoscopic management of gastrointestinal leaks and fistulae. Clin Gastroenterol Hepatol. 2015;13:1714–21. 25697628 10.1016/j.cgh.2015.02.010

[12] Pepe G Chiarello MM Bianchi V. Entero-cutaneous and entero-atmospheric fistulas: insights into management using negative pressure wound therapy. J Clin Med. 2024;13:5. 10.3390/jcm13051279 PMC10932196 38592102

[13] Yanar F Yanar H. Nutritional support in patients with gastrointestinal fistula. Eur J Trauma Emerg Surg. 2011;37:227. 26815104 10.1007/s00068-011-0105-6

[14] Meguid MM Campos AC. Nutritional management of patients with gastrointestinal fistulas. Surg Clin North Am. 1996;76:1035–80. 8841363 10.1016/s0039-6109(05)70497-7

[15] Haffejee AA. Surgical management of high output enterocutaneous fistulae: a 24-year experience. Curr Opin Clin Nutr Metab Care. 2004;7:309–16. 15075923 10.1097/00075197-200405000-00011

[16] Lloyd DA Gabe SM Windsor AC. Nutrition and management of enterocutaneous fistula. Br J Surg. 2006;93:1045–55. 16804873 10.1002/bjs.5396

